# Population Structure and Selection Signatures of Domestication in Geese

**DOI:** 10.3390/biology12040532

**Published:** 2023-03-31

**Authors:** Li Chen, Yongqing Cao, Guoqin Li, Yong Tian, Tao Zeng, Tiantian Gu, Wenwu Xu, Oksana Konoval, Lizhi Lu

**Affiliations:** 1 State Key Laboratory for Managing Biotic and Chemical Threats to the Quality and Safety of Agro-Products, Institute of Animal Science & Veterinary, Zhejiang Academy of Agricultural Sciences, Hangzhou 310021, China; chenli0429@163.com (L.C.);; 2 China-Ukraine Joint Research Center for Protection, Exploitation and Utilization of Poultry Germplasm Resources, Hangzhou 310021, China; 3 Department of Information Technology, National University of Life and Environmental Sciences of Ukraine, 03041 Kiev, Ukraine

**Keywords:** *Anser anser*, *Anser cygnoides*, selection signature, population structure

## Abstract

**Simple Summary:**

The goose is an economically important waterfowl and is one of the first domesticated poultry species. However, population structure and domestication in goose are understudied. In this study, we found that Chinese domestic geese, except Yili geese, originated from a common ancestor and exhibited strong geographical distribution patterns and trait differentiation patterns, while the origin of European domestic geese was more complex, with two modern breeds having Chinese admixture. In both Chinese and European domestic geese, selection signatures during domestication primarily involved the nervous system, immunity, and metabolism, and genes related to vision, skeleton, and blood-O2 transport were also found to be under selection. In particular, we identified that two SNPs in *EXT1* may plausibly be sites responsible for the forehead knob of Chinese domestic geese, and that *CSMD1* and *LHCGR* genes may associate with broodiness in Chinese domestic geese and European domestic geese, respectively. Our study provides new insights into the population structure and domestication of geese.

**Abstract:**

The goose is an economically important poultry species and was one of the first to be domesticated. However, studies on population genetic structures and domestication in goose are very limited. Here, we performed whole genome resequencing of geese from two wild ancestral populations, five Chinese domestic breeds, and four European domestic breeds. We found that Chinese domestic geese except Yili geese originated from a common ancestor and exhibited strong geographical distribution patterns and trait differentiation patterns, while the origin of European domestic geese was more complex, with two modern breeds having Chinese admixture. In both Chinese and European domestic geese, the identified selection signatures during domestication primarily involved the nervous system, immunity, and metabolism. Interestingly, genes related to vision, skeleton, and blood-O2 transport were also found to be under selection, indicating genetic adaptation to the captive environment. A forehead knob characterized by thickened skin and protruding bone is a unique trait of Chinese domestic geese. Interestingly, our population differentiation analysis followed by an extended genotype analysis in an additional population suggested that two intronic SNPs in *EXT1*, an osteochondroma-related gene, may plausibly be sites responsible for knob. Moreover, *CSMD1* and *LHCGR* genes were found to be significantly associated with broodiness in Chinese domestic geese and European domestic geese, respectively. Our results have important implications for understanding the population structure and domestication of geese, and the selection signatures and variants identified in this study might be useful in genetic breeding for forehead knob and reproduction traits.

## 1. Introduction

Animal domestication is a process accompanied by many phenotypic and genetic changes. Detecting the selection signatures underlying domestication is important for understanding the genetic basis of phenotypic changes and will ultimately have enormous practical implications in animal breeding. In recent years, comparative population genomics has identified a number of selective signatures in sheep, chickens, ducks, and other livestock [1,2,3,4,5].

The goose is an economically important waterfowl in the world and is an excellent model for the study of disease resistance and fatty liver because of its low susceptibility to avian viruses and high susceptibility to fatty liver [6]. It is one of the first domesticated poultry: Chinese domestic goose was domesticated over 7000 years ago [7], and European domestic goose was domesticated approximately 5000 years ago [8]. It seems to be an indisputable fact that there are two origins for domestic geese [9,10,11]: Chinese domestic geese (except Yili geese) originate from the swan goose (*Anser cygnoides*), and European domestic geese and Yili geese originate from the greylag goose (*Anser anser*). However, these results are not conclusive because there are still many goose breeds not included in these studies. In fact, the origin of domestic geese is not so straightforward. It has been known that many modern European domestic breeds have admixed background with Chinese domestic goose [12]. Despite the goose’s important and long history of domestication, genome-wide selection signatures during its domestication are still unclear. Compared to their wild ancestors, domestic geese exhibit changes in morphology, behavior, and physiology. For instance, a protuberant knob on the forehead is a prominent characteristic of Chinese domestic geese whereas it is very small or almost absent in their ancestors; meanwhile, both the swan goose and greylag goose exhibit broodiness behavior, but after the long span of domestication, this behavior is absent in some domestic breeds. These changes make the goose a good model for identifying the genetic basis of these phenotypes.

Here, we sequenced whole genomes of geese from two wild ancestral populations, swan goose and greylag goose, five Chinese domestic breeds, and four European domestic breeds to investigate population-level genetic structure and identify selection signatures during goose domestication. Moreover, we employed comparative population genomics to study the genetic basis underlying the forehead knob trait and broodiness behavior.

## 2. Materials and Methods

### 2.1. Sampling and Sequencing

A total of 63 geese representing two wild species, five Chinese domestic breeds, and four European domestic breeds were collected for whole genome resequencing. The five Chinese domestic breeds are typical indigenous breeds: Huoyane goose (HY; *n* = 5), Wulong goose (WL; *n* = 5), Taihu goose (TH; *n* = 5), Lion Head goose (ST; *n* = 5), and Yili goose (YL; *n* = 7). The four European domestic breeds represent the very famous breeds: Roman goose (RM; *n* = 5), Rhine goose (RI; *n* = 5), Sebastopol goose (SV; *n* = 5), and Landaise goose (LD; *n* = 5). These domestic breeds represent various geographic breed origins and phenotypical diversity (Table S1). Samples were also collected from two wild species, the swan goose (SW; *n* = 5) and greylag goose (GR; *n* = 8). Genomic DNA was extracted from blood or feather samples following the standard phenol–chloroform extraction protocol. For each individual, at least 5 mg genomic DNA was used to construct a paired-end library with an insert size of 400 bp according to the manufacturer’s instructions (Illumina, San Diego, CA, USA) and was then sequenced on the Illumina HiSeq platform.

### 2.2. Sequence Mapping and SNP Calling

Filtered reads were mapped to the goose reference genome (GooseV1.0) using BWA-MEM (version 0.7.12-r1039) with default parameters [13]. Sequencing data in SAM files were sorted using SortSam and duplicated reads were removed using the Picard software package (version 1.107). To enhance alignment around indels, sequences were locally realigned using the IndelRealigner tool from the Genome Analysis Toolkit (GATK) (version 3.8) [14]. SNPs were called using the Unified Genotyper implemented in GATK and filtered using the hard filtering process recommended by GATK.

### 2.3. Population Genetic Analysis

PCA based on whole-genome SNPs for all individuals was performed using GCTA v.1.24.2 [15]. A maximum likelihood (ML) phylogenetic tree was built for all samples using RAxML (version 8.2.10) [16]. Population structure analysis was performed using ADMIXTURE (version 1.23) with default settings [17], and the number of assumed genetic clusters ranged from 2 to 10 (K = 2 to 10).

### 2.4. Identification of Divergent Regions

To identify divergent regions between populations, we searched the genome for regions with high *F_ST_* and *θπ* ratio in 40-kb sliding windows with a 10-kb step size using VCFtools [18]. The average *F_ST_* and *θπ* ratio were calculated for the SNPs in each window. Genomic regions with the top 5% *F_ST_* and *θπ* ratio values were considered to be divergent regions.

Functional classification according to GO categories and KEGG pathways was performed using the Database for Annotation, Visualization, and Integrated Discovery (DAVID, v6.8) [19].

### 2.5. Genotype Validation of Candidate Variations

Genotypes of candidate variations were validated in another 62 individuals representing three Chinese indigenous breeds, Zhedong goose, Panshi grey goose, and Yongkang grey goose, and the swan goose. Target variations were amplified using PCR as follows: 5 min at 95 °C; 35 cycles of 95 °C for 30 s, 55 °C for 30 s and 72 °C for 40 s; and a final extension at 72 °C for 5 min. Primers used in the PCR are listed in Table S2. The anticipated PCR bands were purified using a Gel Extraction Kit (Qiagen, Hilden, Germany), and sequenced in 3730XL (Applied Biosystems, Foster City, CA, USA). Finally, results were analyzed using Sequence Scanner software (Applied Biosystems, Foster City, CA, USA).

## 3. Results

### 3.1. Genetic Variation from Genome Resequencing

**Figure 1:**
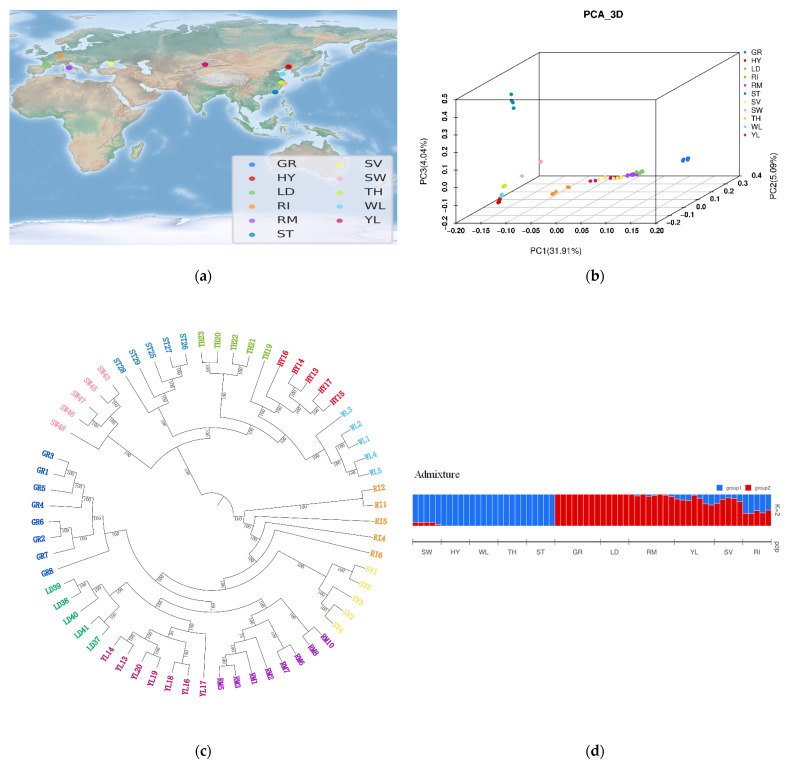
Geographic distribution of breed origins and population structure of domestic and wild geese. (**a**) Geographic distribution of breed origins of the 63 individuals analyzed in this study. (**b**) Phylogenetic tree constructed using all 63 individuals. (**c**) Principal component analysis of the 63 individuals. (**d**) Admixture analysis of the 63 individuals. GR, greylag goose; HY, Huoyane goose; LD, Landaise goose; RI, Rhine goose; RM, Roman goose; ST, Lion Head goose; SV, Sebastopol goose; SW, swan goose; TH, Taihu goose; WL, Wulong goose; YL, Yili goose.

We performed whole−genome resequencing of 63 geese from two wild populations (swan goose and greylag goose), five Chinese domestic breeds, and four European domestic breeds (Figure 1a), with an average coverage depth of ~9.74× for each individual (Table S3). Aligning the reads against the goose reference genome identified a total of 2,505,100 SNPs, with an average of 2.2 SNPs per kilobase. Functional annotation of the SNPs in protein coding regions identified 68,279 (2.73%) nonsynonymous SNPs and 149,646 (5.97%) synonymous SNPs.

### 3.2. Independent Origins of Chinese and European Domestic Geese

To explore the genetic relationships among the 63 individuals, we performed phylogenetic analysis using the maximum likelihood (ML) approach. The phylogenetic tree clearly separated into two clusters: one cluster comprising swan geese and Chinese domestic geese except Yili geese, and the other cluster comprising greylag geese, Yili geese, and European domestic geese (Figure 1b), confirming that European domestic geese and Chinese domestic geese (except Yili geese) were independently domesticated. The non-Yili Chinese domestic geese were further split into two sub-clusters that exhibited strong geographical distribution patterns and trait differentiation. Meanwhile, European domestic geese exhibited more complicated genetic relationships: Landaise geese, Roman geese, and Chinese Yili geese clustered together, separate from greylag geese. Additionally, there were two independent clades: one corresponding to Rhine geese, and other to Sebastopol geese. This phylogenetic pattern was also supported by principal component analysis (PCA) (Figure 1c).

To explore population structure among the 63 individuals, we also conducted a structure analysis by using ADMIXTURE [10]. Partitioning these individuals into two groups gave the K value closest to true (K = 2) (Figure 1d, Figure S1), and clearly separated the samples into: (i) swan geese and non-Yili Chinese domestic geese, which were termed the Chinese group, and (ii) greylag geese, Yili geese, and European domestic geese, which were termed the European group. This is consistent with the results from phylogenetic analysis and PCA.

### 3.3. Independent Selection Signatures in Chinese and European Domestic Geese

In order to detect selection signatures associated with goose domestication, we searched the goose genome for regions with extreme coefficients of nucleotide differentiation (*F_ST_*) and high differences in genetic diversity (*θπ* ratio) between populations of wild and domestic geese. As Chinese domestic geese and European domestic geese are derived from different origins, we analyzed selection signatures in each group separately.

**Figure 2:**
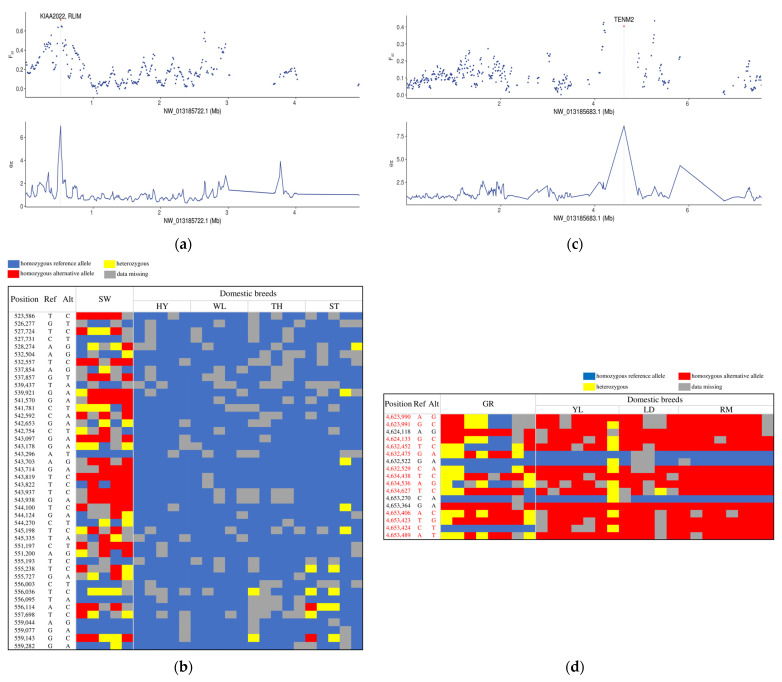
Genomic regions with extreme selection signatures in Chinese domestic geese and European domestic geese. (**a**) *θπ* ratio (*θπ*(wild/domesticated)) and *F_ST_* values along scaffold NW_013185722.1 in the Chinese group. The red dot indicates the region with the highest level of population differentiation. (**b**) Genotypes of 44 SNPs within the highest-differentiation region across swan geese (SW) and four Chinese indigenous breeds (HY, Huoyane goose; WL, Wulong goose; TH, Taihu goose; ST, Lion Head goose). (**c**) *θπ* ratio (*θπ*(wild/domesticated)) and *F_ST_* values along scaffold NW_013185683.1 in the European group. The red dot indicates the region with the highest level of population differentiation. (**d**) Genotypes of 17 SNPs within the highest-differentiation region across greylag geese (GR), Yili goose (YL), and two famous European breeds (LD, Landaise goose; RM, Roman goose). SNPs that showed genetic diversity in greylag geese but were fixed in domestic breeds are listed in red font.

In the Chinese group, a total of 829 regions covering 397 genes were identified as having top 5% *F_ST_* and *θπ* (*θπ*(wild/domesticated)) values and were considered potential selective regions (Table S4, Figure S2). The genomic region NW_013185722.1: 52,001–56,001 stood out as the strongest candidate due to having the highest level of population differentiation (Figure 2a). This region contained 44 SNPs, most of which showed different genotypes between swan geese and domestic breeds (Figure 2b). That is, most of these SNPs showed homozygous mutant genotype in swan geese but were fixed for homozygous reference genotype in all domestic breeds, suggesting this region to have been under hard selection during domestication. The region includes two genes, KIAA2022 and RLIM. KIAA2022 is reportedly associated with the nervous system [20], and RLIM is part of the “Innate Immune System” KEGG pathway.

We selected the top 100 genes with high population differentiation, first selecting the top 50 genes by *F_ST_* values, and then selecting the top 50 genes by *θπ* ratio without overlapping with genes selecting using *F_ST_* method. Annotation of the top 100 genes revealed over-representation of functions associated with metabolism, immunity, and the nervous system (Table 1). It is worth noting that we also observed enrichment of genes functionally related to vision, the skeleton, and the hematological system. Functional enrichment analysis of all the 397 genes using Gene Ontology and KEGG identified over-representation of GO terms related to the nervous system and behavior, along with one KEGG pathway associated with reproduction (Table S5).

In selection analysis of the European group, Rhine geese and Sebastopol geese were excluded due to those breeds comprising independent clades. In total, 736 putative selective regions covering 494 genes were identified as having top 5% values for both *F_ST_* and *θπ* ratio (Table S6, Figure S2). The strongest candidate region (NW_013185683.1: 4,620,001–4,660,001) was found within the gene Teneurin transmembrane Protein 2 (*TENM2*) (Figure 2c), which has been reported to control brain development and neuronal wiring [21]. This region contained 17 SNPs, of which 13 presented genetic diversity in greylag geese but were fixed in the domestic breeds (Figure 2d).

Inspection of the top 100 selected genes with high population differentiation, selecting using the method described above, revealed similar enriched categories of gene function as in the Chinese group. That is, metabolism, immunity, and the nervous system were the primary functional categories, and genes associated with bone development, vision, and hematopoiesis were also over-represented (Table 2). Functional enrichment analysis of these 494 genes revealed significant enrichment for GO terms involved in the nervous system, hemostasis, and muscle development (Table S6). Meanwhile, pathway analysis identified over-representation of three pathways, neuroactive ligand–receptor interaction, starch and sucrose metabolism, and calcium signaling (Table S7).

Comparative analysis of candidate genes between Chinese and European groups identified only 22 genes shared by the two groups. These genes had functions associated with immunity, metabolism, nervous development, growth, and reproduction (Table S8).

### 3.4. Selection Signatures Controlling Protuberant Knob

**Figure 3:**
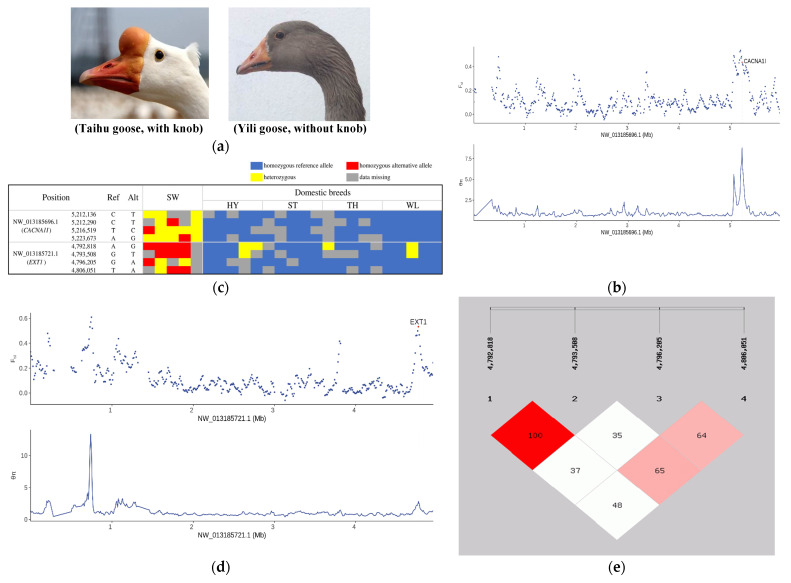
Selection signatures controlling the protuberant knob trait. (**a**) Different phenotypes of knob. (**b**) *θπ* ratio (*θπ*(wild/domesticated)) and *F_ST_* values around *CACNA1I*. (**c**) SNPs that presented genotype differentiation between Chinese domestic breeds and their wild counterpart, the swan goose. (**d**) *θπ* ratio (*θπ*(wild/domesticated)) and *F_ST_* values around *EXT1*. (**e**) Linkage analysis based on four candidate SNPs.

Compared to their wild ancestors, Chinese domestic goose other than Yili goose has a protuberant knob on the forehead (Figure 3a). To identify candidate genes responsible for this trait, we inspected 397 genes selected in Chinese domestic breeds, of which two candidate genes caught our attention. The first was calcium voltage-gated channel subunit alpha1 I (*CACNA1I*), which was in the top 0.5% for both *F_ST_* and *θπ* ratio values (Figure 3b). *CACNA1I* is an important paralog of *CACNA1H*, which was previously reported to relate to protuberant knob in geese [22]. In the genomic region of *CACNA1I*, we identified four SNPs, three intronic and one exonic, that exhibit genotype differentiation between Chinese domestic breeds and their wild counterpart, the swan goose (Figure 3c). However, all four SNPs were excluded as candidate sites because their genotypes did not segregate with the phenotype when examined in another 62 individuals representing swan geese and three indigenous Chinese breeds (Table S9), suggesting that *CACNA1I* is not in fact associated with the protuberant knob trait. The other candidate gene was an osteochondroma-related gene, Exostosin glycosyltransferase 1 (*EXT1*), which also showed a relatively high level of population differentiation (Figure 3d). Allele frequency analysis of all SNPs in the selected region where *EXT1* was located revealed that there were 15 SNPs with significant differences in allele frequency between Chinese domestic breeds and swan geese (Table S10). Genotype screening of these 15 SNPs identified four intronic SNPs in *EXT1* to present genotype differentiation between populations (Figure 3c). Linkage analysis of the four SNPs revealed that two SNPs (NW_013185721.1: 4,792,818 and 4,793,508) were in complete linkage disequilibrium (LD, r2 = 1.0; Figure 3e). Genotype analysis of the four SNPs in another 62 individuals revealed the two linked SNPs to have perfect genotype segregation with protuberant knob (Table 3, Table S9), suggesting that these two SNPs may be associated with the trait. Finally, from among the 397 candidate genes, another four selected genes, *DIO3*, *PDGFD*, *TSHR*, and *FRZB*, were previously identified to be associated with knob [22,23]. These mutations and genes provide candidates for genetic discovery of the protuberant knob trait in geese.

### 3.5. Genetic Signatures Related to Broodiness Behavior

**Figure 4:**
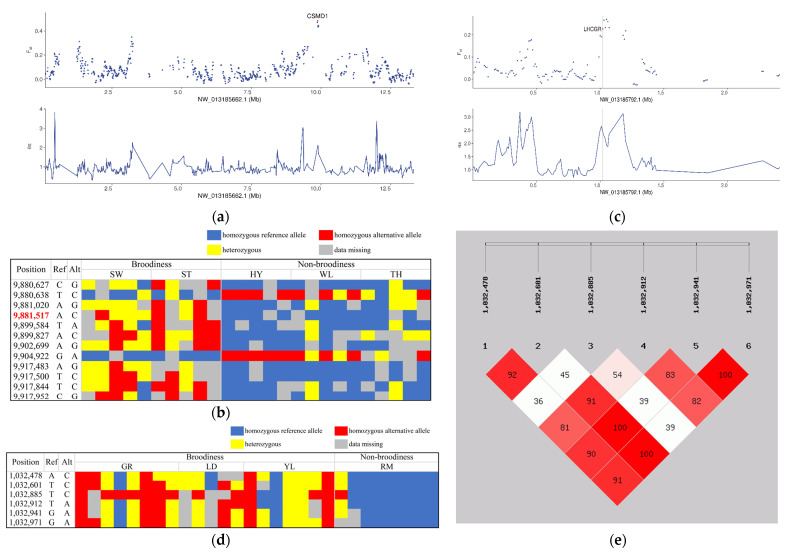
Genetic signatures related to broodiness behavior. (**a**) *θπ* ratio (*θπ*(broodiness/non-broodiness)) and *F_ST_* values around *CSMD1*. (**b**) Genotypes of 22 SNPs within *CSMD1* in broody and non-broody populations. SNP that presented genotype differentiation between populations was listed in red font. (**c**) *θπ* ratio (*θπ*(broodiness/non-broodiness)) and *F_ST_* values around *LHCGR*. (**d**) The 8.2-kb region showing differentiation of genetic diversity between broody and non-broody populations. (**e**) Linkage analysis based on six candidate SNPs.

To identify genetic signatures associated with broodiness behavior, we separately searched Chinese and European goose genomes for regions with high *F_ST_* and *θπ* ratio between populations exhibiting broodiness and non-broodiness. In the Chinese group, this analysis highlighted 695 regions covering 438 genes (Table S11). The highest level of population differentiation was observed for the region NW_013185662.1: 9,880,001–9,920,001, which contained 22 SNPs and was sited within the gene CUB and Sushi Multiple Domains 1 (*CSMD1*) (Figure 4a), previously implicated in chicken egg production [24].

Allele frequency analysis of all the 22 SNPs revealed that there were 12 SNPs with significant differences in allele frequency between populations (Table S12). Genotype analysis of the 12 SNPs indicated an A to C intronic mutation (NW_013185662.1: 9,881,517) which displayed perfect genotype segregation with the phenotype (Figure 4b). Additionally, the gene follicle-stimulating hormone receptor (*FSHR*), which was previously reported to be associated with broodiness behavior, also showed differentiation between broody and non-broody populations (Table S11).

In the European group, signature analysis identified 461 regions covering 326 genes that showed a high level of population differentiation (Table S13). The region with the highest degree of differentiation overlapped with the luteinizing hormone/choriogonadotropin receptor (*LHCGR*) gene (Figure 4c), which is an important paralog of *FSHR*; coincidentally, *FSHR* was also identified as a candidate gene for this phenotype (Table S13). Allele frequency analysis of all SNPs in this region revealed that there were six SNPs with significant differences in allele frequency between populations (Table S14). Genotype analysis of the six SNPs identified that they presented genetic diversity in broody populations but were almost fixed in non-broody populations (Figure 4d). Linkage analysis of the six SNPs revealed that three SNPs (NW_013185792.1: 1,032,601, 1,032,941 and 1,032,971) were in complete linkage disequilibrium (LD, r2 = 1.0; Figure 4e), which suggest that they may candidate variations for broodiness behavior but this still needs further validation.

## 4. Discussion

The goose was one of the first domesticated poultry species, and is still economically important. In this study, we sequenced whole genomes of 63 geese from two wild populations, five Chinese domestic breeds, and four European domestic breeds, explored the population structure and domestication of Chinese and European domestic geese, and further explored genes associated with broodiness behavior and a protuberant forehead knob. Our study provides important implications for understanding the population structure and domestication of geese.

Our population genetic analysis and selection analysis show that Chinese and European domestic geese are two separate groups, providing genomic evidence that Chinese domestic geese and European domestic geese were derived from different origins. Chinese domestic geese other than Yili geese are genetically closely related to swan geese, while Yili geese and two European domestic geese (Landaise geese and Roman geese) are genetically close to greylag geese; these findings suggest that Chinese domestic geese (except Yili) may originate from swan geese while Yili geese, Landaise geese, and Roman geese may originate from greylag geese. This is supported by previous population analyses [9,10,11]. In the European group, we note that the Rhine goose had almost half admixed background with Chinese domestic geese, and it constituted a separate population but was genetically closely related to Chinese domestic breeds. The Rhine goose is an improved breed developed by the French Creamer company. We speculate that the Chinese domestic goose may have been introduced in the earliest formation of the Rhine goose, but it is also possible that the Rhine goose was crossed with Chinese domestic geese after being introduced to China. After all, the samples we collected came from populations introduced to China, not from the breed’s country of origin. We noted that Sebastopol geese also constituted a separate population and had admixed background with Chinese domestic geese, confirming that this breed is a hybrid between Chinese and European domestic geese, consistent with a previous study [12].

By contrasting domestic with wild samples, we identified 397 and 494 candidate genes that are under selection in Chinese and European groups, respectively. Functional annotation of the top 100 candidate genes revealed the nervous system as the most over-represented functional category associated with domestication in both groups. In particular, strong selection signatures located in or within *KIAA2022* and *TENM2* implied intense selection relating to the nervous system. Selection signatures for the nervous system have been observed in many species, such as sheep, dingoes, and ducks [2,3,4], indicating that the nervous system is the first to be affected during domestication, leading domesticated animals to exhibit prosocial behaviors. In addition, immunity and metabolism were also identified as primary functional categories subjected to selection. Selection signatures for metabolism and immunity have been observed in other animals such as sheep, dogs, and ducks [1,3,25]. This may relate to adaptation to a new environment in the forms of diet and immune system changes.

It is worth noting that a few selected genes were found to correlate to vision, the skeleton, and blood-O2 transport. Bird flight demands a high rate of O2 consumption; as an extreme example, the O2 consumption of bar-headed geese steadily flying in a wind tunnel at sea level ranges from 10- to 15-fold above resting levels [26]. Evolution of genes involved in blood-O2 transport in support of environmental adaption has been well documented in animals living in hypoxic high-altitude areas [27,28]. After being domesticated, geese live in a captive environment, leading to the most prominent of their phenotypic changes, namely, loss of flight ability. That genes involved in blood-O2 transport, such as *BACH1*, *ABCB7*, and *HBS1L*, are under selection in domestic geese suggests a genetic adaptation to the captive environment, which may be an adaptation to the loss of flight ability.

Similarly, the process of domestication results in significant morphological changes to the skeleton, with key examples being a decline in skeletal robusticity, reduction in cranial size, shortening of limbs, reduction in molar size, and changes in body size [29]. In geese, domestication has caused larger body size, stronger leg bones, and shorter and broader wing bones [30]. In this study, genes related to the skeleton such as *PAPPA2*, *TRAPPC3L*, *WWOX*, and *NT5DC1* were found to have undergone selection in domestic geese. This may be correlated with adaptation to the captive environment or directional selection by humans for body size.

Compared to their wild ancestors, domestic animals exhibit many phenotypic changes, but a particularly interesting one is their comparatively weaker vision. Markedly weaker visual acuity relative to wild ancestors has been reported in dogs, horses, and chickens [31,32,33]. Like other domestic animals, domestic geese also harbor reduced visual acuity as compared to the swan goose or greylag goose. In this study, vision-related genes including *APAF1*, *GRIK1*, and *ALDH8A1* were found to be under selection in domestic geese, which might have contributed to their reduced visual acuity.

A knob on the forehead is a prominent trait of Chinese domestic geese, whereas it is very small or almost absent in swan geese and absent in greylag geese and European domestic geese [34]. It is characterized by thickened skin and protruding bone, and its size mainly depends on the breed, age, and sex of the goose. Morphology of the cranial appendage is tightly correlated with the physiology and reproduction of animals [35,36]. For example, Shelducks with large knobs have more advantages in competing for mates and territorial protection [37]. In chicken, rose-comb was found to be associated with reduced male fertility [35]. In production, a goose with a large knob seems to exhibit a higher social rank, better health status, and higher breast muscle weight [34]. Therefore, a well-developed knob is preferred by customers and has become one of the main breeding targets for geese in China. However, in stark contrast with the popularity of the knob phenotype, little is known about its genetic basis. In this work, we identified through population differentiation analysis that an osteochondroma-related gene, *EXT1*, exhibits a relatively high level of population differentiation between Chinese domestic geese and swan geese. Further genotype analysis in an expanded population revealed two *EXT1* SNPs (at positions 4,792,818 and 4,793,508 in scaffold NW_013185721.1) to show genotype segregation with the knob trait. In humans, *EXT1* has been linked to tricho-rhino-phalangeal syndrome (TRPS) and multiple osteochondromas (MO) [38,39]; TRPS is characterized by skeletal and craniofacial abnormalities, and MO is characterized by multiple cartilage-capped bony outgrowths of the long bones, resulting in a variety of complications such as skeletal deformity. Skeletal abnormality is also observed in the goose knob, which is obviously protruding. Therefore, the two SNPs may plausibly be sites responsible for the trait, and may be useful in genetic breeding for this trait.

Broodiness behavior seriously affects egg production. To identify genes associated with this economically important trait, we performed comparative population genomics in the Chinese group and European group separately. In the Chinese group, we found *CSMD1* to show the highest level of differentiation between broody and non-broody populations. This gene has been proposed to relate to reproduction; *Csmd1* knockout in mice reduced fertility through altered regulation of spermatozoa production [40]. In the chicken, *CSMD1* is considered potentially related to egg production [41]. Interestingly, we found an intronic mutation (NW_013185662.1: 9881517, A < C) in *CSMD1* that displayed perfect genotype segregation with broodiness behavior. However, there is still need of further correlation between this SNP and the phenotype. Meanwhile, in the European group, an 8.2-kb region in *LHCGR* exhibited the highest differentiation between broody and non-broody populations. *LHCGR* is an important paralog of *FSHR*, a G-protein coupled receptor for follicle-stimulating hormone that plays a major role in reproduction; loss of its function results in pronounced disturbance of spermatogenesis and folliculogenesis [42,43]. It is very interesting that *FSHR* also showed differentiation between broody and non-broody populations in both the Chinese group and European group, suggesting that this gene may correlate with broodiness behavior. *FSHR* has been previously reported to correlate with broodiness in the chicken.

## 5. Conclusions

In this study, whole genome resequencing of geese from two wild populations, five Chinese domestic breeds, and four European domestic breeds was performed. It is the first selection analysis of geese domestication at the genome-wide level. Chinese domestic geese originate from a common ancestor, while the origin of European domestic geese was more complex, with two modern breeds having Chinese admixture. We also discovered many selection signatures of domestication, which primarily involved the nervous system, immunity, and metabolism. In particular, two intronic SNPs in *EXT1* were found to be possibly associated with knob, and *CSMD1* and *LHCGR* genes may associate with broodiness in Chinse domestic geese and European domestic geese, respectively. Collectively, these findings provide new insights into the population structure and domestication of geese, and the selection signatures and variants identified in this study might be useful in genetic breeding for forehead knob and reproduction traits.

## Figures and Tables

**Table 1:** Metabolism, immunity and nervous system-related genes among the top 100 selected genes in Chinese domestic geese.

Scaffold	Start (bp)	End (bp)	*θπ* Ratio (Wild/Domestic)	*F_ST_*	Gene Name	Functions
NW_013185722.1	520,001	560,001	7.02084	0.714427	*RLIM*	Innate immune system pathway
NW_013185722.1	520,001	560,001	7.02084	0.714427	*KIAA2022*	Involved in neurite outgrowth
NW_013185659.1	5,240,001	5,280,001	5.69171	0.555266	*AKT3*	Hippocampal neurogenesis
NW_013185696.1	5,050,001	5,090,001	5.6146	0.492042	*RPS19BP1*	Cellular responses to stimuli
NW_013185696.1	5,180,001	5,220,001	5.57682	0.536577	*CACNA1I*	Involved in sensory processing, sleep, and hormone and neurotransmitter release
NW_013185664.1	5,530,001	5,570,001	5.48137	0.47688	*LPIN1*	Involved in lipid metabolism
NW_013185657.1	7,890,001	7,930,001	4.82417	0.480043	*PTGR2*	Arachidonic acid metabolism
NW_013185662.1	12,000,001	12,040,001	4.62785	0.48803	*DLGAP2*	Plays a role in synapse organization and signaling in neuronal cells
NW_013185657.1	7,910,001	7,950,001	4.4634	0.493049	*UBR1*	Class I MHC mediated antigen processing and presentation pathway
NW_013185706.1	4,210,001	4,250,001	4.30241	0.644791	*ANKS1B*	Brain development
NW_013185706.1	4,210,001	4,250,001	4.30241	0.644791	*APAF1*	Visual system
NW_013185654.1	22,740,001	22,780,001	4.0863	0.572681	*KAT6B*	Involved in cerebral cortex development
NW_013185779.1	1,580,001	1,620,001	4.04274	0.362452	*PAPPA2*	Regulates bone structure and mass
NW_013185885.1	180,001	220,001	4.00233	0.444173	*HTR1D*	Affects neural activity
NW_013185716.1	70,001	110,001	3.98051	0.441914	*LDLRAP1*	Lipid metabolism
NW_013185722.1	480,001	520,001	3.58249	0.636686	*SLC16A2*	Development of central nervous system
NW_013185930.1	710,001	750,001	3.4615	0.45944	*BEGAIN*	Regulates postsynaptic neurotransmitter receptor activity
NW_013185657.1	8,100,001	8,140,001	3.31629	0.63377	*TTBK2*	Involved in atrophy of the cerebellum and brainstem
NW_013185657.1	8,130,001	8,170,001	3.13448	0.695921	*STARD9*	Lipid binding
NW_013185657.1	8,130,001	8,170,001	3.13448	0.695921	*CDAN1*	Essential for primitive erythropoiesis
NW_013185662.1	11,990,001	12,030,001	3.06677	0.490442	*CLN8*	Involved in neuronal differentiation
NW_013185654.1	6,470,001	6,510,001	2.99721	0.610978	*PAX2*	Participates in optic nerve development
NW_013185885.1	130,001	170,001	2.93483	0.510357	*LUZP1*	Affects neural tube
NW_013185657.1	8,230,001	8,270,001	2.92731	0.597566	*SNAP23*	Class I MHC mediated antigen processing and presentation pathway
NW_013185662.1	11,980,001	12,020,001	2.92009	0.469008	*ARHGEF10*	Neural morphogenesis
NW_013185663.1	10,900,001	10,940,001	2.83154	0.520603	*NCOA2*	Circadian clock pathway; Glucose metabolism regulation
NW_013185779.1	1,550,001	1,590,001	2.61207	0.494237	*RFWD2*	Class I MHC mediated antigen processing and presentation pathway
NW_013185657.1	9,070,001	9,110,001	2.55479	0.512538	*TYRO3*	Innate immune response; Neuron protection
NW_013185799.1	2,270,001	2,310,001	2.45909	0.493535	*BACH1*	Heme binding
NW_013185672.1	7,580,001	7,620,001	2.35532	0.475914	*HBS1L*	Controls fetal hemoglobin level
NW_013185672.1	7,580,001	7,620,001	2.35532	0.475914	*ALDH8A1*	Visual system
NW_013185666.1	8,860,001	8,900,001	2.30898	0.484818	*PI4KA*	Neurodevelopment
NW_013185741.1	2,060,001	2,100,001	2.22717	0.450407	*DHRS3*	Visual phototransduction pathway
NW_013185799.1	2,230,001	2,270,001	2.19869	0.566629	*GRIK1*	Involved in transmission of light information
NW_013185722.1	2,650,001	2,690,001	2.1901	0.584116	*SMARCA1*	Promotes brain development
NW_013185657.1	9,010,001	9,050,001	1.92798	0.56243	*MAPKBP1*	Immune function
NW_013185885.1	80,001	120,001	1.84796	0.657325	*KDM1A*	Involved in blood cell development
NW_013185661.1	4,790,001	4,830,001	1.81422	0.484537	*JAZF1*	Glucose and lipid metabolism
NW_013185657.1	8,260,001	8,300,001	1.72594	0.577736	*ZNF106*	Essential for skeletal muscle function
NW_013185769.1	770,001	810,001	1.69181	0.517866	*FOXP2*	Neurodevelopment
NW_013185657.1	7,400,001	7,440,001	1.68652	0.481765	*DPF3*	Plays an essential role in heart and skeletal muscle development
NW_013185722.1	700,001	740,001	1.68606	0.446956	*ABCB7*	Involved in the transport of heme
NW_013185682.1	300,001	340,001	1.5411	0.478458	*SHPRH*	Metabolism of proteins pathway
NW_013185746.1	1,540,001	1,580,001	1.52872	0.478827	*NLGN4X*	Remodels central nervous system synapses

**Table 2:** Metabolism, immunity, and nervous system related genes among the top 100 selected genes in European domestic geese.

Scaffold	Start (bp)	End (bp)	*θπ* Ratio (Wild/Domestic)	*F_ST_*	Gene Name	Functions
NW_013185673.1	20,001	60,001	1.7753	0.573003	*TRAPPC3L*	Bone development
NW_013185676.1	6,660,001	6,700,001	7.19466	0.456279	*WWOX*	Bone development
NW_013185673.1	200,001	240,001	3.73375	0.230746	*NT5DC1*	Bone development
NW_013185673.1	230,001	270,001	3.52744	0.380911	*COL10A1*	Bone development
NW_013185655.1	5,790,001	5,830,001	2.06836	0.416861	*HMX1*	Development
NW_013185859.1	1,260,001	1,300,001	1.77307	0.431281	*BLMH*	Immunity
NW_013185855.1	1,150,001	1,190,001	4.47664	0.322131	*EDA2R*	Immunity
NW_013185882.1	440,001	480,001	1.70704	0.44064	*BANP*	Immunity
NW_013185655.1	16,110,001	16,150,001	3.93205	0.381584	*BCL11B*	Immunity
NW_013185714.1	560,001	600,001	1.84461	0.413279	*SLC25A5*	Metabolism
NW_013185676.1	3,500,001	3,540,001	3.6863	0.236796	*FAM96B*	Metabolism
NW_013185718.1	2,100,001	2,140,001	3.60565	0.280045	*MCAT*	Metabolism
NW_013185718.1	2,100,001	2,140,001	3.60565	0.280045	*TSPO*	Metabolism
NW_013185718.1	2,100,001	2,140,001	3.60565	0.280045	*TTLL12*	Metabolism
NW_013185673.1	30,001	70,001	2.01444	0.48736	*DSE*	Metabolism
NW_013185667.1	9,680,001	9,720,001	4.9104	0.391882	*XDH*	Metabolism
NW_013185667.1	9,870,001	9,910,001	3.73004	0.253108	*GALNT14*	Metabolism
NW_013185656.1	15,270,001	15,310,001	2.0786	0.410677	*GBE1*	Metabolism
NW_013185683.1	4,620,001	4,660,001	8.61059	0.405009	*TENM2*	Nervous system
NW_013185656.1	17,300,001	17,340,001	7.02891	0.241569	*ROBO2*	Nervous system
NW_013185659.1	14,670,001	14,710,001	4.59723	0.278432	*PRKCE*	Nervous system
NW_013185792.1	190,001	230,001	4.44657	0.35268	*ALK*	Nervous system
NW_013185660.1	4,120,001	4,160,001	3.69867	0.261144	*SLC4A10*	Nervous system
NW_013186039.1	90,001	130,001	3.64631	0.340177	*FLOT2*	Nervous system
NW_013185810.1	920,001	960,001	3.50133	0.403726	*EXOC2*	Nervous system
NW_013185725.1	2,390,001	2,430,001	3.37828	0.281384	*TRAPPC6B*	Nervous system
NW_013185725.1	2,390,001	2,430,001	3.37828	0.281384	*GEMIN2*	Nervous system
NW_013185656.1	10,860,001	10,900,001	0.459251	0.459251	*EPHA6*	Nervous system
NW_013185745.1	430,001	470,001	1.74703	0.433555	*GRM8*	Nervous system
NW_013185859.1	1,260,001	1,300,001	1.77307	0.431281	*SLC6A4*	Nervous system
NW_013185702.1	3,680,001	3,720,001	2.04116	0.42798	*SEMA5A*	Nervous system
NW_013185677.1	5,000,001	5,040,001	4.49648	0.375977	*DNAH3*	Reproduction
NW_013185676.1	6,370,001	6,410,001	4.84849	0.467322	*ADAMTS18*	Visual system
NW_013185868.1	780,001	820,001	4.031	0.220905	*OPTN*	Visual system
NW_013186039.1	90,001	130,001	3.64631	0.340177	*ERAL1*	Growth
NW_013185664.1	7,050,001	7,090,001	7.54069	0.219905	*NBAS*	Growth; Visual system
NW_013185703.1	1,950,001	1,990,001	2.17876	0.42861	*JAG1*	Hematopoiesis

**Table 3:** Genotype segregation of four candidate variations in *EXT1* with the protuberant knob phenotype in different populations.

Breed/Species	N	Segregation of Genotype with Phenotype (Yes/No)			
		4,792,818	4,793,508	4,796,205	4,806,051
Zhedong goose	17	17/0	17/0	17/0	13/4
Panshi grey goose	18	18/0	18/0	18/0	18/0
Yongkang grey goose	20	20/0	20/0	20/0	10/10
Swan goose	7	7/0	7/0	5/2	2/5
**Total**	**62**	**62/0**	**62/0**	**60/2**	**43/19**

N = number of individuals.

## Data Availability

Not applicable.

## Supplementary Materials

The following supporting information can be downloaded at: https://www.mdpi.com/article/10.3390/biology12040532/s1. Whole genome resequencing data have been submitted to the SRA database in NCBI with the BioProject accession number PRJNA911405.
