# Supplementary material for: Population Structure and Selection Signatures of Domestication in Geese
Source: Biology (Basel). 2023 Mar 31;12(4):532. doi: 10.3390/biology12040532 (PMC10136318; doi:10.3390/biology12040532)

Figure S1: Structure analysis of 63 individuals using ADMIXTURE (version 1.23) with the number of assumed genetic clusters ranging from 2 to 10 (K = 2 to 10). (a) Distribution of CV values corresponding to K values. The K value that corresponds to the smallest CV value is the true K value. (b) Population structure with K values ranging from 2 to 10.

a **b**

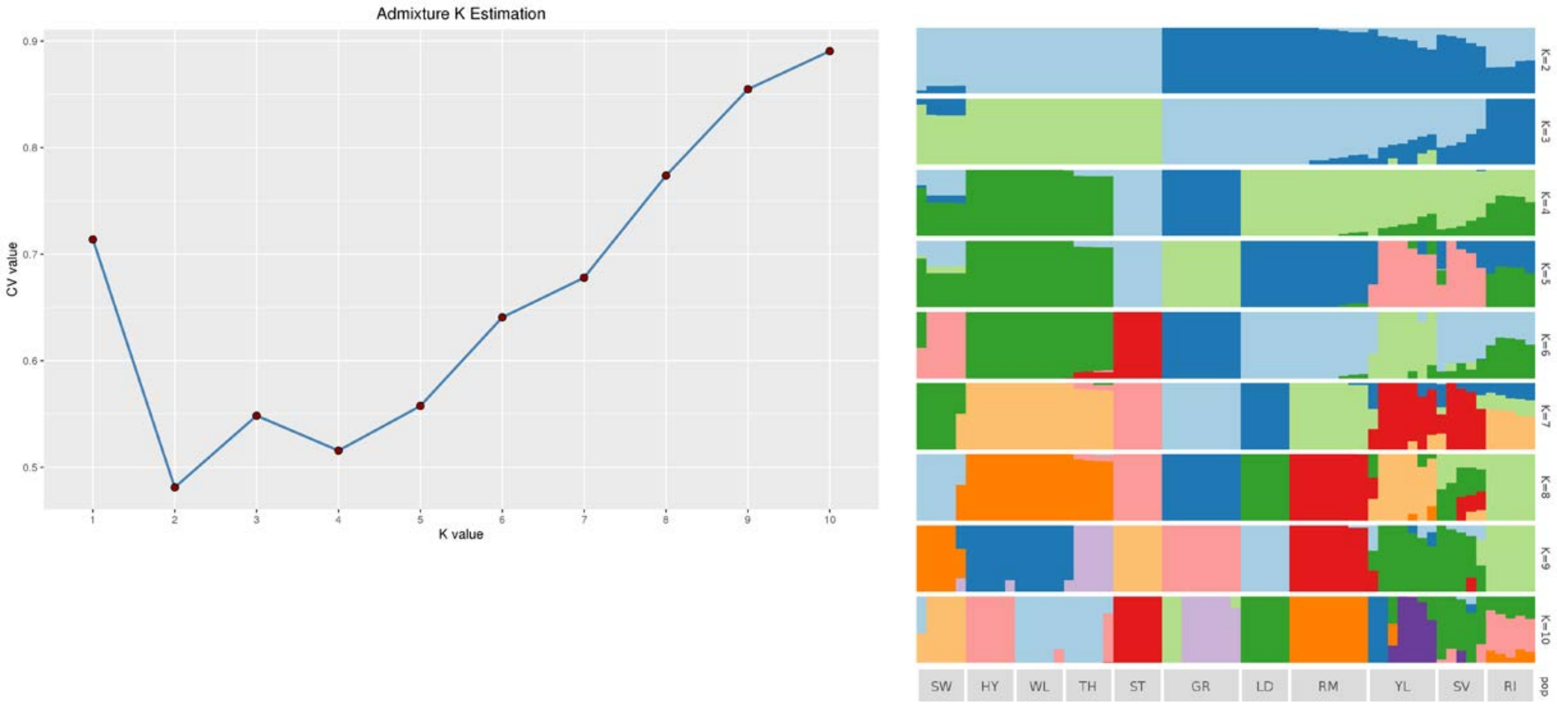

Supplement: Supplementary file 1 [file biology-12-00532-s001.zip › biology-2192236-supplementary/10. FS1.pdf]
