# Supplementary material for: Population Structure and Selection Signatures of Domestication in Geese
Source: Biology (Basel). 2023 Mar 31;12(4):532. doi: 10.3390/biology12040532 (PMC10136318; doi:10.3390/biology12040532)

Figure S2. Genome-wide distribution of the *θπ* ratio and F_{ST}, determined in 40-kb windows. (a) Between swan geese and Chinese domestic breeds (except Yili geese). (b) Between greylag geese and European domestic breeds (including Yili geese but excluding Rhine and Sebastopol geese). Red dots represent regions fulfilling the requirement for selection. Vertical and horizontal gray dashed lines represent the thresholds of *θπ* ratio and F_{ST}, respectively.

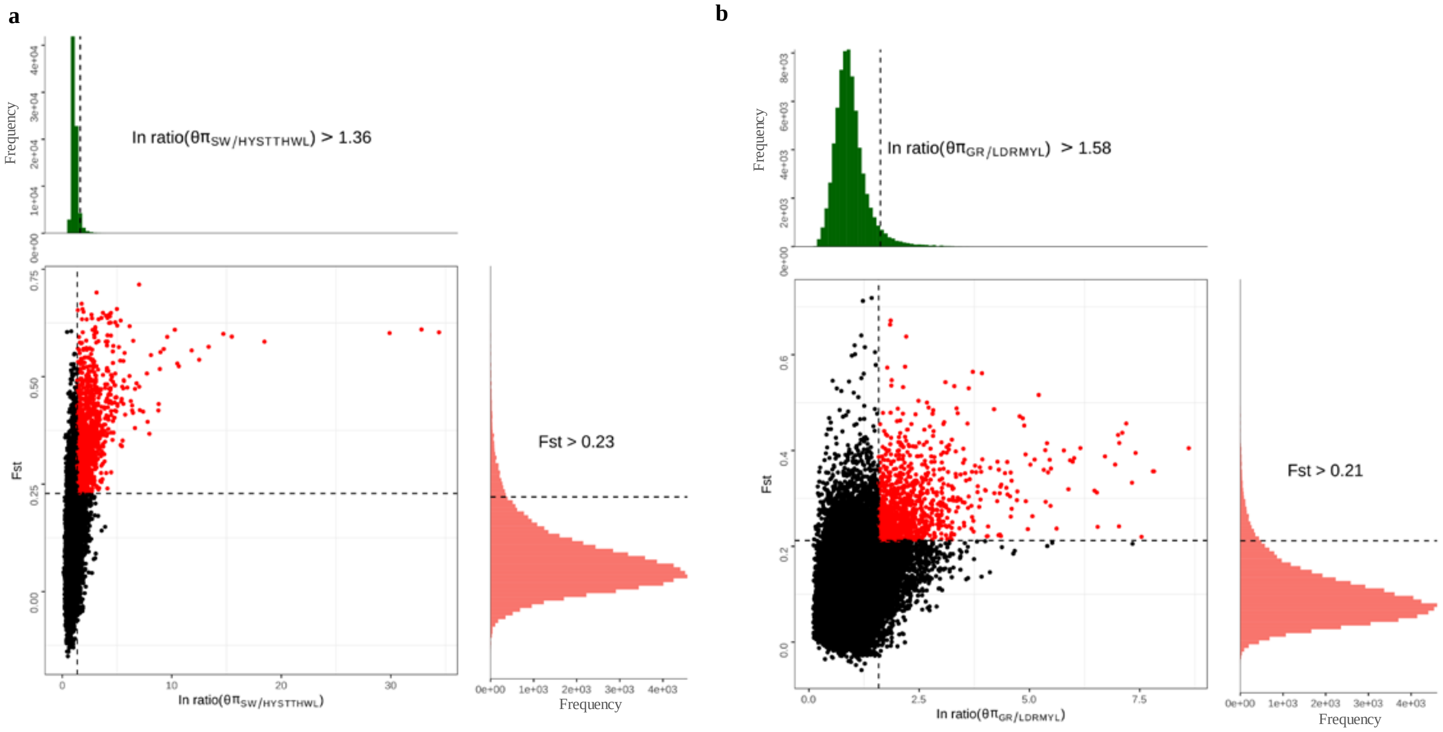

Supplement: Supplementary file 1 [file biology-12-00532-s001.zip › biology-2192236-supplementary/11.FS2.pdf]
