# Supplementary material for: Population Structure and Selection Signatures of Domestication in Geese
Source: Biology (Basel). 2023 Mar 31;12(4):532. doi: 10.3390/biology12040532 (PMC10136318; doi:10.3390/biology12040532)
Supplement: Supplementary file 1 [file biology-12-00532-s001.zip › biology-2192236-supplementary/Table S1.pdf]

Table S1. Breed information for two wild ancestral populations, five Chinese domestic breeds, and four European domestic breeds.

Breeds/Species	Appearance	Characteristics	Distribution
Swan goose	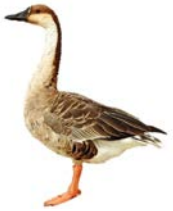	• Wild type<br>• Small body size<br>• Broody<br>• Very little forehead knob	Distributed in China, southern Siberia, and Central Asia.
Huoyane goose	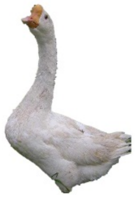	• Chinese indigenous breed<br>• Small body size<br>• Non-broody<br>• With forehead knob	This breed originated in Tieling, Liaoning Province, China.
Wulong goose	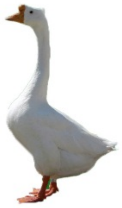	• Chinese indigenous breed<br>• Small body size<br>• Non-broody<br>• With forehead knob	This breed originated in Laiyang, Shandong Province, China.
Taihu goose	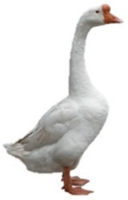	• Chinese indigenous breed<br>• Small body size<br>• Non-broody<br>• With forehead knob	This breed is native to area along the Taihu Lake.
Lion Head goose	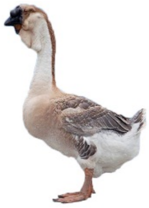	• Chinese indigenous breed<br>• Large body size<br>• Broody<br>• With large forehead knob	This breed originated in Chaozhou City, Guangdong Province, China.
Greylag goose	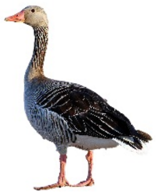	• Wild type<br>• Small body size<br>• Broody<br>• Without forehead knob	Distributed in northern Europe, Siberia, Central Asia, and China.
Roman goose	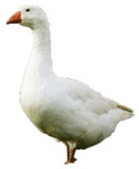	• Famous European domestic breed<br>• Non-broody (or rarely broody)<br>• Middle body size<br>• Without forehead knob	This breed originated in Italy and was introduced to Taiwan.

Rhine goose	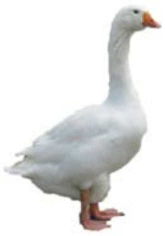	• Famous European domestic breed<br>• Non-broody<br>• Middle body size<br>• Without forehead knob	Rhine geese are native to the Rhine valley of Germany and are bred by the French Creamer company.
Yili goose	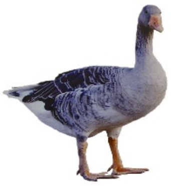	• Chinese indigenous breed<br>• Broody<br>• Middle body size<br>• Without forehead knob	This breed originated in Yili, Xinjiang Province, China.
Landaise goose	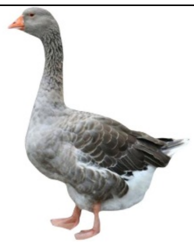	• Famous European domestic breed<br>• Broody<br>• Large body size<br>• Without forehead knob	The breed is native to Landes, southwest France, near the Bay of Biscay.
Sebastopol goose	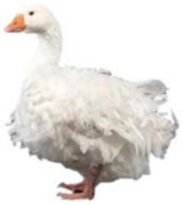	• Famous European domestic breed<br>• Non-broody<br>• Middle body size<br>• Without forehead knob	The Sebastopol goose originated in southeastern Europe, with sources pointing to the region around the Black Sea.
