# Supplementary material for: Population Structure and Selection Signatures of Domestication in Geese
Source: Biology (Basel). 2023 Mar 31;12(4):532. doi: 10.3390/biology12040532 (PMC10136318; doi:10.3390/biology12040532)
Supplement: Supplementary file 1 [file biology-12-00532-s001.zip › biology-2192236-supplementary/Table S2.pdf]

Table S2. Primers used in genotyping of the candidate variations

SNP marker	Primer sequence (5’-3’)
NW_013185721.1:<br>4,792,818	F: CTGTAGACGCTGGGATGTAA<br>R: GATCTCGCAGACAGTTATACAC
NW_013185721.1:<br>4,793,508	F: CACCACAAACCCTTTGAGCG<br>R: GGACAAACGCCCAGGTAAA
NW_013185721.1:<br>4,796,205	F: TTAACCGGTCGCAGTCTTGT<br>R: ACGTGAAGGCTGAGGTGTTGG
NW_013185721.1:<br>4,806,051	F: CCCGTGTAGACCATACCTCA<br>R: ACACCTGCTTCTTCCCGTG
NW_013185696.1:<br>5,212,136	F: ATGGCTGGGGCTGTAATCTG<br>R: TGGGTTAGGATCTGCAACCAG
NW_013185696.1:<br>5,212,290	F: ATGGCTGGGGCTGTAATCTG<br>R: TGGGTTAGGATCTGCAACCAG
NW_013185696.1:<br>5,216,519	F: AGTCTGCGGCCTGAAACAG<br>R: TGCGGTTGTTCATCTTTGGG
NW_013185696.1:<br>5,223,673	F: CCTTTGGGTCAAGGCTTTGC<br>R: TCAGGTACAACCCCAATACGTG
