# Supplementary material for: Population Structure and Selection Signatures of Domestication in Geese
Source: Biology (Basel). 2023 Mar 31;12(4):532. doi: 10.3390/biology12040532 (PMC10136318; doi:10.3390/biology12040532)
Supplement: Supplementary file 1 [file biology-12-00532-s001.zip › biology-2192236-supplementary/Table S8.pdf]

Table S7. Candidate selected genes shared by the Chinese and European group.

Gene symbol	LOC106033043, LOC106045569, MBTD1, STXBP4, ALCAM, BCKDHB, CA10, CTNNA3, CXADR, EDA2R, EPHA6, GBE1, INHBB, LOC106036557, LOC106039940, MC2R, NBAS, PARK2, SMYD3, TBC1D32, TOM1L1, UTP18
